# Bridging immunization gaps: lessons from Zambia’s 2024 measles–rubella supplementary immunisation activity

**DOI:** 10.3389/fpubh.2025.1625514

**Published:** 2025-07-03

**Authors:** Moses Mwale, Penelope Masumbu, Peter Jay Chipimo, Patrick Sakubita, Andrew Phiri, Kennedy Matanda, Kelvin Mwangilwa, Andrea Carcelen, Simon Mutembo, Freddie Masaninga, Jackob Sakala, Peter Clement Lugala

**Affiliations:** ^1^World Health Organization, Lusaka, Zambia; ^2^The Ministry of Health, Lusaka, Zambia; ^3^The Zambia National Public Health Institute, Lusaka, Zambia; ^4^International Vaccine Access Center, Johns Hopkins Bloomberg School of Public Health, Baltimore, MD, United States

**Keywords:** Zambia immunization, measles-rubella campaign, zero-dose children, community engagement, digital monitoring, supplementary immunization, low-resource settings

## Abstract

Zambia’s 2024 Measles-Rubella Supplementary Immunization Activity (SIA), conducted from 23 to 28 September across all 116 districts, targeted children aged 9–59 months to address immunization gaps exacerbated by COVID-19 disruptions and responding to ongoing measles outbreaks. This community case study evaluates the effectiveness of microplanning, the feasibility of real-time digital monitoring, and the equity of reaching zero-dose children during Zambia’s 2024 Measles-Rubella SIA, using a mixed-method approach to inform scalable immunization strategies in resource-limited settings. Through comprehensive microplanning, strategic community engagement, and real-time digital monitoring, the campaign achieved 97% national coverage and reached 165,000 previously zero-dose children in underserved communities. Implementation utilized Google Sheets and Open Data Kit tools, with quality assurance through over 7,500 supervisory visits. Despite achieving high overall coverage, several challenges emerged: funding delays, logistical constraints in remote areas, and data quality issues. Key lessons include the necessity of timely funding disbursement, strengthened cold chain infrastructure, and rigorous data verification processes. Community involvement through local leadership engagement and radio campaigns proved essential to success, while digital monitoring enabled rapid adaptation to emerging challenges. This case study provides actionable insights for designing equitable immunization campaigns in resource-limited settings, supporting global measles and rubella elimination goals through evidence of effectively tailored, data-driven strategies.

## 1 Introduction

Measles and rubella remain significant global health challenges despite the availability of effective vaccines (1, 2). In 2023, the World Health Organization (WHO) reported 9.8 million measles cases and approximately 107,500 deaths, with low-income countries bearing the highest burden due to vaccination coverage as low as 64% (3, 4). The COVID-19 pandemic exacerbated these gaps, reducing global first-dose measles-containing vaccine (MCV1) coverage to 81% in 2021, with only modest recovery to 83% by 2022 (5). This decline has fueled outbreaks, particularly in regions with disrupted immunization services, underscoring the need for targeted supplementary immunization activities (SIAs) to achieve the 95% coverage required for herd immunity (6).

**Figure 1:**
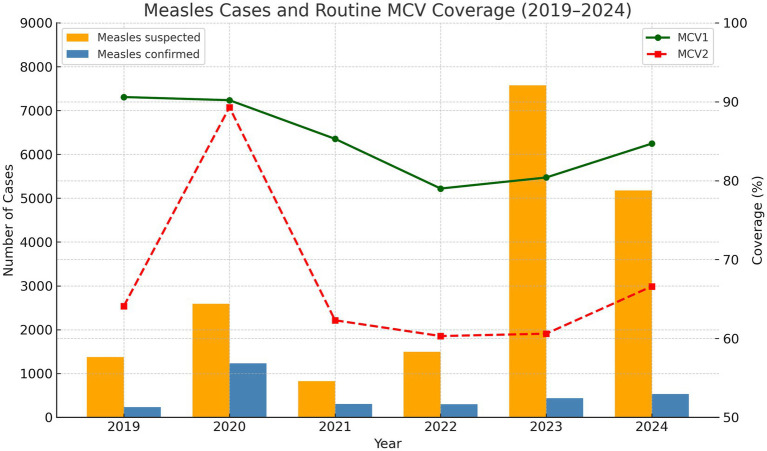
Routine MCV1 and MCV2 administrative coverage rates by year 1998 to 2024. Number of suspected measles and confirmed cases from 2019 to 2024.

In Zambia, measles and rubella continue to threaten child health despite progress in routine immunization. Since introducing combined measles-rubella (MR) vaccination in 2016, Zambia has improved population immunity, but only 52 of 116 districts achieve the recommended 95% MCV1 coverage (7, 8). The situation deteriorated further during the COVID-19 pandemic, which severely disrupted immunization services (9, 10) (Figure 1).

The pandemic’s impact resulted in nearly 500,000 Zambian children missing their first measles vaccine dose between 2020 and 2023, with approximately 300,000 receiving neither the first nor second dose (11). This accumulation of unvaccinated (“zero-dose”) children has facilitated renewed transmission, with over 3,429 cases reported in between January 2020 to August 2023 (10). In 2022, the country recorded over 420 confirmed measles cases with 37 deaths, with multiple districts experiencing prolonged outbreaks (12). These immunity gaps, particularly concentrated in underserved and remote communities, present an urgent public health challenge: without intensified immunization efforts, Zambia faces continued outbreaks that endanger child health (13). Responding to WHO’s Measles and Rubella Strategic Framework 2021–2030 (6), Zambia’s Ministry of Health, in collaboration with implementing partners, conducted the 2024 Measles-Rubella SIA from 23 to 28 September. The campaign targeted 4 million children aged 9–59 months across all 116 districts, employing microplanning, strategic community engagement, and real-time digital monitoring to reach zero-dose children and achieve equitable coverage. This community case study evaluates the effectiveness of microplanning and digital monitoring in achieving equitable immunization coverage during Zambia’s 2024 Measles–Rubella Supplementary Immunization Activity (SIA). It documents strategies, outcomes, and operational barriers, providing actionable insights for designing resilient SIAs and strengthening routine immunization in Zambia and similar low-resource settings, contributing to global measles and rubella elimination goals. Data were gathered through a systematic review of program documents, aggregation of routine immunization data, and real-time digital monitoring reports.

## 2 Key programmatic elements

### 2.1 Planning and coordination

Planning and Coordination Zambia initiated planning for the 2024 Measles-Rubella Supplementary Immunization Activity (MR SIA) 6 months prior to its 23–28 September rollout, engaging stakeholders at national, provincial, and district levels to ensure operational precision. Health facilities developed detailed microplans delineating catchment zones, estimating target populations (children aged 9–59 months), and identifying potential vaccination sites. These plans were systematically consolidated at district and provincial levels, harmonizing population estimates, schedules, and resource requirements before submission to the national program office.

Comprehensive readiness assessments evaluated planning, coordination, service delivery, monitoring, cold chain logistics, and advocacy efforts, ensuring all critical components for successful campaign execution were in place. These district-level assessments identified infrastructural and operational gaps—ranging from refrigeration capabilities to transport and staffing limitations—that could potentially affect vaccine delivery. Insights gained from these assessments guided the strategic allocation of vaccines and resources, ensuring necessary support was directed to areas with greatest need.

**Figure 2:**
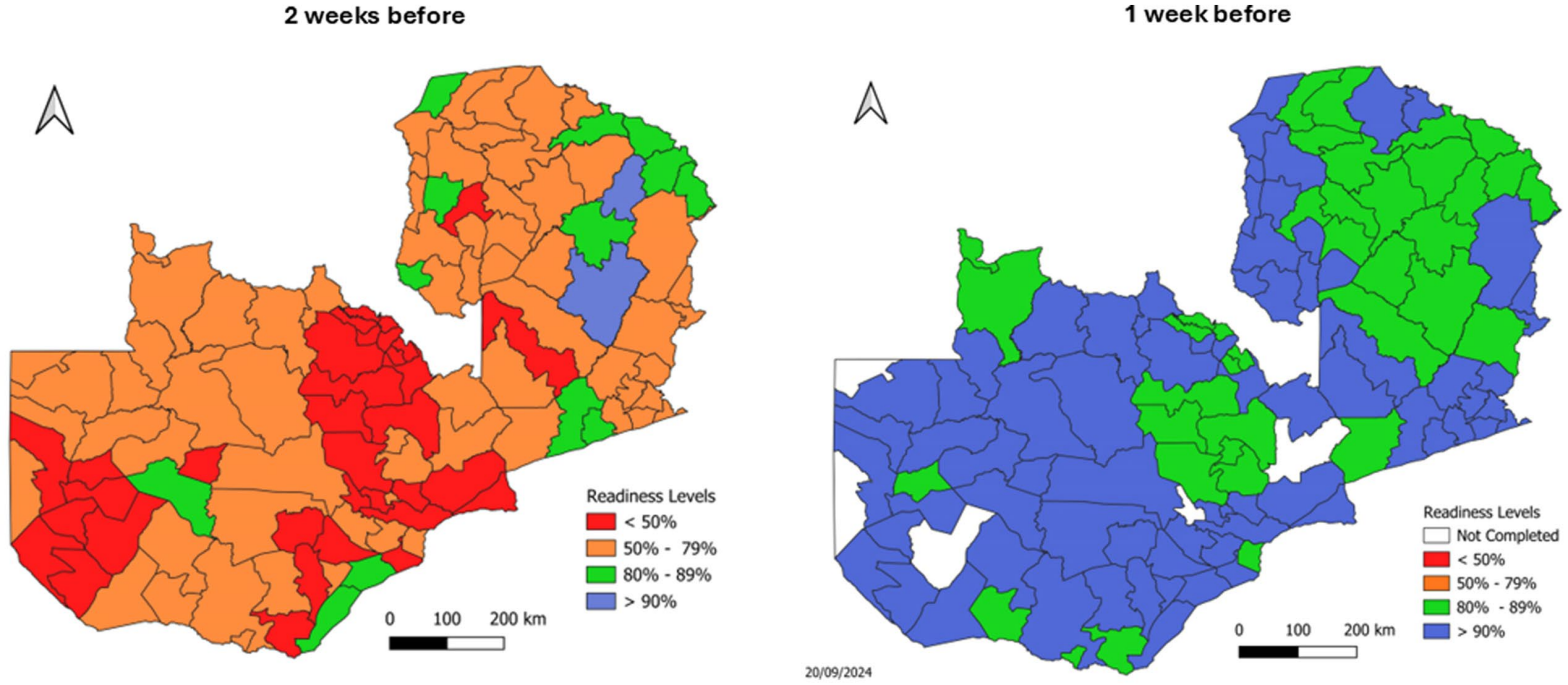
Subnational readiness assessments for Zambia’s 2024 MR SIA, highlighting improvements from 2 weeks before (left) to 1 week before (right) the campaign.

This bottom-up approach grounded national strategies in local realities, informing vaccine procurement, mobile team deployment, and training priorities. Two weeks before the campaign, a final evaluation (Figure 2, left map) confirmed that targeted interventions—including expedited funding, redistributed training guides, and completed monitor trainings—had addressed most identified gaps. One week prior to launch (Figure 2, right map), increased transport availability and training completion shifted most districts to high readiness status (green/blue areas), enabling rapid reallocation of remaining resources. This data-driven planning approach optimized resource utilization and strengthened coordination mechanisms, laying a robust foundation for the MR SIA’s success.

### 2.2 Community engagement and social mobilization

Community engagement proved pivotal to the MR SIA’s success. District-level stakeholder meetings secured active participation pledges from local chiefs, religious leaders, and community influencers. In Southern Province, for example, community leaders provided transportation and water supplies for health workers. Trained community health workers and volunteers conducted systematic door-to-door mobilization, informing households about vaccination schedules and locations.

Local radio stations offered up to 2 h of daily free airtime for campaign jingles and announcements, while public address systems reached densely populated urban areas. These combined interpersonal and mass media efforts ensured broad awareness, significantly boosting turnout in remote and traditionally underserved communities and enhancing vaccine delivery efficiency.

### 2.3 Human resource mobilization and training

The MR SIA mobilized 11,016 health workers and 18,421 volunteers nationwide, training them through a cascade model to ensure campaign readiness. National and provincial experts trained district-level trainers via in-person and virtual workshops, who subsequently equipped local health workers and volunteers with necessary skills.

Training comprehensively covered vaccine administration techniques, adverse events following immunization (AEFI) management protocols, digital data collection tools, and broader immunization topics such as cold chain maintenance and medical waste disposal. This systematic approach ensured consistent skills transfer across all 116 districts, significantly enhancing service delivery quality and overall campaign capacity.

### 2.4 Vaccine logistics and cold chain management

The campaign procured and distributed over 4.3 million vaccine doses nationwide. Pre-campaign assessments of refrigeration, freezer, and cold box capacities identified potential gaps, prompting targeted solutions like additional ice packs and portable cold boxes in Eastern Province to counter frequent power outages.

A digital inventory system enabled real-time stock monitoring at distribution points, facilitating rapid responses to emerging shortages. This integrated approach maintained vaccine potency throughout the supply chain, ensuring efficient delivery to even the most remote immunization posts.

### 2.5 Data collection, supervision, and real-time monitoring

Vaccination sites utilized standardized Google Sheets forms to capture daily data on age groups (9–11, 12–23, 24–59 months), vaccine stocks, and alerts (AEFI, suspected measles cases, refusals), enabling real-time coverage tracking. Supervision teams employed Open Data Kit (ODK) to collect systematic data on eligibility screening, injection techniques, and vaccine sufficiency, with geolocation features enhancing oversight capabilities. To ensure data quality, Google Sheets templates incorporated validation rules to flag inconsistent entries, such as age or dose mismatches, while ODK forms used skip logic and mandatory fields to reduce errors. Training for data collectors emphasized accurate tallying and error-checking protocols. Daily virtual feedback meetings with district supervisors reviewed submissions for completeness and accuracy, resolving discrepancies through phone calls or follow-up visits. However, network connectivity issues occasionally delayed data submission.

Over 7,000 supervisory visits, peaking at 1,782 on Day 2, were conducted across all districts, supported by daily virtual feedback meetings. These digital tools and established feedback loops allowed rapid identification and resolution of emerging issues like transport delays, fostering an adaptive campaign approach that ultimately drove high coverage rates.

## 3 Results

### 3.1 Coverage and vaccination outcomes

**Figure 3:**
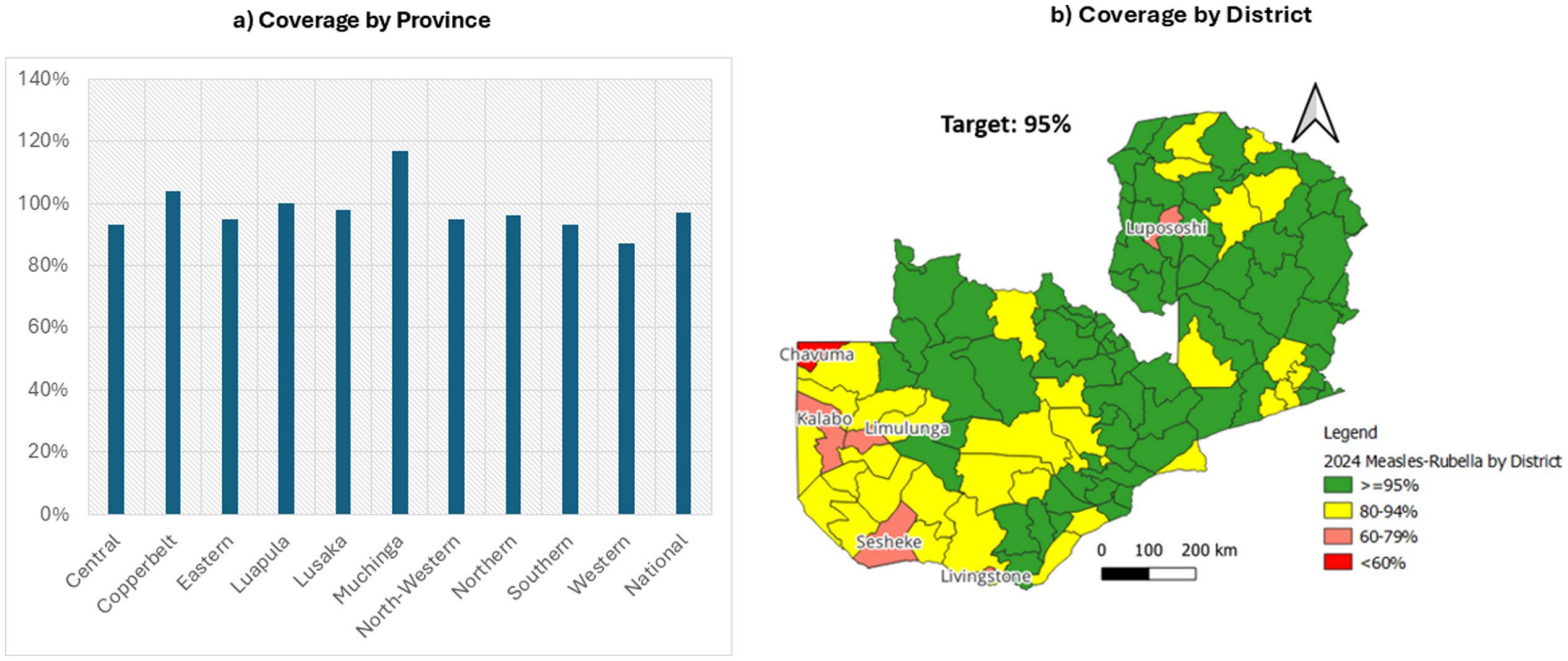
Provincial and district coverage of Zambia’s 2024 measles-rubella SIA (19).

The 2024 MR SIA campaign achieved a national administrative coverage of 97%. Several provinces surpassed the 95% target—including Muchinga (117%), Eastern (104%), and Copperbelt (105%)—while Central (93%) and Western (87%) fell short. At the district level, most areas achieved ≥ 95 per cent coverage; however, Chavuma (Northwestern Province) recorded below 60 per cent, and Kalabo, Limulunga and Sesheke (all in Western Province) fell in the 60–79 per cent range. Livingstone (Southern Province) attained 80–94 per cent. Coverage rates exceeding 100% in provinces like Muchinga, Eastern, and Copperbelt likely reflect inaccuracies in target population estimates or potential duplicate reporting during high-mobility campaign days. These discrepancies highlight limitations in administrative data reliability and underscore the need for updated demographic data and robust verification processes in future campaigns. Figure 3 presents provincial (left) and district-level (right) measles-rubella SIA coverage rates.

Age-specific data revealed that older children (24–59 months) closely approached their vaccination targets, whereas the 9–11 months cohort exhibited a more pronounced shortfall. Gender distribution was nearly equitable, with 53% of vaccinated children being female and 47% male. Despite these achievements, approximately 165,000 children aged 9–59 months were identified as zero-dose recipients—emphasizing the campaign’s critical role in addressing immunity gaps among hard-to-reach populations. Fishing communities in Muchinga and Luapula, migrant populations in Southern Province, and remote villages in Western Province emerged as particular areas of concern, underscoring the importance of continued targeted interventions to ensure equitable immunization coverage (Table 1).

**Table 1:** Target and vaccinated children by province and age group in the 2024 measles-rubella SIA, Zambia.

Province	9–11 months			12–23 months			24–59 months			9–59 months		
	Tgt	Vac	Cov (%)	Tgt	Vac	Cov (%)	Tgt	Vac	Cov (%)	Tgt	Vac	Cov (%)
Central	29,809	48,866	163.9	138,196	161,519	116.9	409,061	304,065	74.4	552,787	514,450	93.1
Copperbelt	23,901	39,857	166.8	111,505	141,723	127.1	330,055	282,228	85.5	446,019	463,808	104.0
Eastern	29,362	36,960	126.0	132,546	156,618	118.2	392,337	310,998	79.3	530,186	504,576	95.2
Luapula	20,253	32,541	160.7	91,385	119,915	131.3	270,494	212,542	78.6	365,534	364,998	99.9
Lusaka	28,734	72,411	252.1	133,390	175,478	131.6	394,834	275,145	69.7	533,559	523,034	98.0
Muchinga	10,221	17,260	168.9	45,253	64,211	142.0	133,949	130,381	97.3	181,013	211,852	117.0
North Western	17,738	25,342	142.8	68,374	80,346	117.6	198,935	166,752	83.8	285,049	272,440	95.6
Northern	19,013	33,445	175.9	87,081	104,773	120.3	257,759	197,153	76.5	348,323	335,371	96.3
Southern	24,749	34,643	140.0	116,033	124,647	107.4	343,458	273,142	79.5	464,132	432,432	93.2
Western	18,034	29,195	161.9	85,246	91,341	107.1	252,328	177,140	70.2	340,984	297,676	87.3
Grand Total	221,814	370,520	167.0	1,009,009	1,220,571	120.9	2,983,210	2,329,546	78.1	4,047,586	3,920,637	96.9

Tgt, Target; Vac, Vaccinated; Cov (%), Coverage (Percentage).

### 3.2 Operational achievements

The campaign demonstrated significant operational successes through robust community engagement and extensive human resource mobilization. Building on established Ministry of Health structures, the campaign efficiently coordinated a nationwide network of 16,136 operational immunization posts, 6,838 vaccination teams, 5,600 supervisors, and 18,421 volunteers. This large-scale deployment, facilitated by close coordination among government agencies, NGOs (WHO, CIDRZ, UNICEF, CHAZ), and local communities, enabled effective navigation of complex logistical challenges.

More than 28,000 health workers and volunteers were trained through a cascade model that empowered district-level trainers to build capacity locally. This comprehensive training enhanced vaccine administration techniques, AEFI management, and digital data collection proficiency. Detailed microplans allowed districts to tailor strategies to local contexts, optimizing resource allocation and intensifying supervision in areas with initial low coverage. Daily supervisory visits supported by digital monitoring tools facilitated real-time corrective actions, contributing significantly to the overall quality of vaccination sessions.

### 3.3 Monitoring and supervision outcomes

Across all 116 districts, 7,057 supervisory visits were conducted at static and outreach sites, demonstrating high compliance with key vaccination-quality standards (Table 2). Quality of Vaccination Practice indicators achieved 100% compliance in most areas, including injection technique, dose tallying, reconstitution, and child positioning, with hand-washing or sanitizer availability marginally lower at 96%. In Logistics & Cold-chain, vaccine sufficiency was 100%, while emergency tray and AEFI reporting form availability reached 93 and 91%, respectively. Community Awareness & Data Tools indicators, including accurate dose recording, community mobilization, and monitoring-tool use, exceeded 97% compliance.

**Table 2:** Vaccination site compliance by category.

Category	Indicator	Sites meeting standard (%)
Quality of vaccination practice	AD syringes & cotton-wool availability	100%
	Correct injection technique	100%
	Correct marking with indelible markers	100%
	Correct reconstitution demonstration	100%
	Hand-washing or sanitizer availability	96%
	MR vaccines packaged properly	99%
	Proper child positioning	100%
	Screening for eligibility	100%
	Sharp-box availability	100%
Logistics & cold-chain	AEFI reporting forms available	91%
	Emergency tray available	93%
	Vaccines sufficient today	100%
Community awareness & data tools	Accurate recording of MR doses	100%
	Community awareness methods	100%
	Local personnel included	100%
	MR tallying appropriately	99%
	Public aware of SIA dates	99%
	Tally-sheets & monitoring tools	97%

Post-visit reports nonetheless revealed persistent gaps in hygiene, emergency preparedness, and vaccine supply. Many sites lacked soap, sanitizer, or running water, and teams occasionally overlooked hand-washing kits. Emergency trays were similarly neglected—either through human error, stock-outs of key drugs, or transport challenges—and some staff were unaware of their necessity at outreach points. Vaccine shortages arose when district allocations or cold-chain delays failed to meet revised targets.

### 3.4 Challenges encountered

Despite overall high administrative coverage, several operational challenges emerged. Funding delays in districts (particularly Central, Copperbelt, Lusaka, and Muchinga) hindered timely preparation, including training and resource mobilization. Logistical constraints, such as shortages of transport, fuel, and vehicles, limited vaccination team reach, especially in rural areas of Western and North-Western provinces. Cold chain management issues persisted, with inadequate refrigeration, limited cold boxes, and frequent power outages in Muchinga and Southern provinces threatening vaccine potency.

Environmental challenges, including droughts and seasonal flooding, necessitated unconventional transport methods (boats, oxcarts) to access remote communities. Data reporting was hampered by network connectivity issues, delaying real-time submissions and affecting monitoring accuracy.

The 2024 MR-SIA reported 124 adverse events following immunization (AEFIs), primarily in Southern (60 cases) and Copperbelt (20 cases) provinces, with 115 minor and 9 serious cases, mostly linked to MCV2 (92.8%). Minor AEFIs, affecting males slightly more (55.6%), included fever (72 cases), skin rash (24 cases), and diarrhea (19 cases). Serious AEFIs comprised anaphylaxis (2 cases), febrile seizures (3 cases), and 4 cases with incomplete documentation, likely due to time constraints and non-standardized forms. This gap limited safety analysis, necessitating standardized AEFI reporting forms and enhanced training for future campaigns.

The absence of rapid convenience monitoring surveys, due to logistical constraints, prevented real-time coverage validation, relying instead on administrative data prone to discrepancies. These challenges highlight the need for improved resource allocation, infrastructure, and data systems to enhance campaign effectiveness.

## 4 Discussion

The 2024 MR SIA campaign demonstrated that a well-coordinated, data-driven approach can yield high coverage even in challenging environments. The campaign’s success was largely attributable to detailed district-specific microplans, intensive social mobilization, a cascade training model for over 11,000 health workers and nearly 18,500 volunteers, and real-time digital supervision tools for monitoring and reporting. Monitoring results showing total compliance in injection technique, dose tallying, reconstitution, and child positioning, with only marginal shortfalls in hand-washing (96%) and AEFI form availability (91%), confirm the effectiveness of cascade training and real-time supervision while pinpointing specific areas for targeted improvement. Despite these successes, funding delays, logistical constraints related to transport and fuel, and persistent cold chain gaps, particularly in Muchinga and Southern provinces, moderated overall impact. Although routine MCV1/MCV2 coverage in 2019–2020 exceeded levels observed in 2024, confirmed case counts were higher in 2020. This suggests that, despite stronger routine performance before the COVID-19 pandemic, the 2024 SIA’s intensive, campaign-driven approach was necessary to bridge immunity gaps.

At the district level, several areas fell below the national goals. Chavuma (Northwestern Province) recorded coverage below 60 per cent, a deficit largely attributable to geographic isolation, seasonal flooding of the Zambezi floodplain and limited road access. In Western Province, Kalabo, Limulunga and Sesheke each reported coverage in the 60–79 per cent range. These districts share Barotse floodplain terrain, where seasonal inundation and poor roads impeded timely outreach, leading to recurrent cold-chain breaches and delayed engagement with community leaders. In Southern Province, Livingstone achieved 80–94 per cent coverage; high population mobility driven by cross-border commerce and tourism complicated household enumeration and resulted in pockets of under-immunized children. These low-performing districts highlight the necessity of context-specific adaptations—such as pre-positioning vaccines before peak flooding, strengthening transport contingency plans and engaging transient populations—to ensure equitable coverage in future campaigns.

Zambia’s 2024 MR-SIA significantly outperformed the 2020 MR-SIA, which achieved 68.6% coverage amidst COVID-19 restrictions and reached only 27.8% of measles zero-dose children (21). The 2024 campaign’s 97% coverage and vaccination of 165,000 zero-dose children resulted from refined microplanning and digital tools like Google Sheets and Open Data Kit, which enabled rapid adaptations absent in 2020’s restricted context. By contrast, India’s 2017–2019 MR-SIA fell below 95% in studied districts due to logistical challenges and vaccine hesitancy (14), and Malawi’s 2017 MR-SIA reached only 59% in Lilongwe due to low awareness (15). Like Nigeria’s polio SIAs, which used mobile technology for high coverage (16), Zambia’s 2024 digital innovations highlighted tailored strategies’ value for global measles and rubella elimination. These comparisons highlight Zambia’s strengths in achieving high coverage and equity through tailored strategies, offering valuable lessons for global immunization efforts aiming to meet measles and rubella elimination goals.

### 4.1 Best practices

The 2024 MR campaign demonstrated several best practices that significantly contributed to its success. District-specific microplanning combined with rigorous supervision and real-time monitoring allowed rapid identification and resolution of service delivery gaps. Integrating SIA microplans into routine planning ensured previously missed communities were captured, strengthening overall system resilience. The benefits of real-time digital supervision in improving data quality have been observed in prior studies (17).

The cascade training approach ensured consistent knowledge transfer and capacity building at every administrative level. Active community engagement through local leaders, community-based volunteers, door-to-door sensitization, church announcements, and tailored mobilization strategies substantially improved turnout, even in hard-to-reach areas. Effective community engagement has proven critical in similar campaigns internationally (18).

Innovative adaptations enhanced the campaign’s reach; mobile vaccination teams employed various transportation methods, including boats, motorbikes, and oxcarts, to access isolated fishing camps and remote villages, demonstrating flexibility and the importance of customized approaches to meet local conditions.

### 4.2 Areas for improvement

To strengthen future immunization campaigns, several areas require attention. First, ensuring timely funding disbursement is critical to avoid delays in preparation and training. Establishing pre-campaign funding protocols can mitigate this issue. Second, enhancing transport logistics through expanded vehicle fleets and fuel reserves will improve access to remote areas. Third, addressing cold chain gaps demands investment in reliable refrigeration and backup power solutions, particularly in high-risk provinces. Fourth, optimizing digital infrastructure is essential to overcome network connectivity issues, ensuring seamless real-time data reporting. Fifth, incorporating rapid convenience monitoring surveys can validate coverage estimates and guide timely mop-up activities. Finally, improving AEFI reporting by training health workers to document event details will enhance vaccine safety monitoring. These targeted improvements can bolster operational efficiency and equity in immunization delivery across Zambia and similar settings.

### 4.3 Implications for future immunization strategies

Future immunization strategies should integrate detailed microplanning, intensive social mobilization, cascade training models, and real-time digital supervision into routine programs. Equally critical is establishing robust transportation networks, as reliable transport underpins every aspect of vaccine delivery—from distribution to supervisory visits and mop-up activities.

Future campaigns must rigorously address infrastructural and logistical gaps identified in pre-campaign readiness assessments to ensure corrective measures are fully implemented before launch. Strengthening funding mechanisms and guaranteeing timely resource allocation remain vital for sustaining high performance, with this campaign’s demonstrated cost-effectiveness serving as a benchmark for future budgeting.

Policymakers should invest in essential infrastructure, particularly enhancements to cold-chain systems, transportation logistics, and digital reporting platforms, to bolster operational responsiveness and build resilient immunization strategies.

### 4.4 Policy implications

Based on these outcomes, actionable recommendations for policymakers and public health practitioners emerge. First, establishing effective funding protocols and increasing investments in transport, cold chain systems, and data reporting tools are essential. Expanding the cascade training model to include refresher courses for monitors and refining microplanning strategies to create dedicated vaccination points in both urban and remote areas will help ensure no community is left behind.

Enhanced communication strategies through local media, community leaders, and religious institutions can address information gaps and reduce vaccine hesitancy. Finally, investing in digital infrastructure for real-time data collection, reporting, and surveillance will enable prompt, informed responses, ultimately strengthening routine immunization programs. Implementing these recommendations will optimize resource allocation, enhance campaign performance, and contribute to sustainable, equitable vaccine coverage across Zambia and similar low-resource settings.

### 4.5 Limitations

This study relies on program data, which may overestimate administrative coverage (97% nationally) due to inaccurate target population estimates or reporting errors, as evidenced by coverage rates exceeding 100% in some provinces. Rapid convenience surveys (RCS) were not conducted during the 2024 MR-SIA due to financial limitations that restricted resources for additional fieldwork. Instead, monitoring teams conducted 7,057 supervisory visits and a post-campaign coverage survey (PCCS) was planned to estimate community-level coverage and validate administrative data. The absence of RCS may have delayed identification of unvaccinated children in districts like Chavuma (72%) and Kalabo (87%; Table 1), necessitating future funding for rapid surveys. Network connectivity issues delayed real-time data submission in some districts, potentially affecting monitoring accuracy. Lastly, the absence of qualitative data from communities or health workers limits insights into barriers to uptake. These limitations underscore the need for improved data verification, digital infrastructure, and qualitative research in future campaigns. Future campaigns should also include independent statistical oversight to enhance data credibility.

## 5 Conclusion

Zambia’s 2024 Measles-Rubella Supplementary Immunization Activity offers a model for addressing immunization gaps in low-resource settings, achieving high coverage through innovative strategies like microplanning, community engagement, and real-time digital supervision. By reaching underserved populations, including 165,000 zero-dose children, the campaign advanced equitable vaccine access and aligned with global measles and rubella elimination goals (20).

However, challenges such as funding delays, logistical constraints, and data inaccuracies highlight the need for enhanced infrastructure and verification processes. Policymakers and public health practitioners should prioritize investments in cold chain systems, transport logistics, and digital platforms to build resilient immunization programs.

Sustained collaboration among government, NGOs, and communities is essential to integrate these lessons into routine systems, ensuring no child is left behind. Zambia’s experience provides actionable insights for other low-resource settings, demonstrating that adaptive, data-driven approaches can overcome complex barriers to achieve sustainable, equitable immunization coverage, ultimately contributing to healthier populations worldwide.

## Data Availability

The raw data supporting the conclusions of this article will be made available by the authors, without undue reservation.
